# Supplementary material for: First Report of blaNDM-1 Bearing IncX3 Plasmid in Clinically Isolated ST11 Klebsiella pneumoniae from Pakistan
Source: Microorganisms. 2021 Apr 28;9(5):951. doi: 10.3390/microorganisms9050951 (PMC8146611; doi:10.3390/microorganisms9050951)
Supplement: Supplementary file 1 [file microorganisms-09-00951-s001.zip › microorganisms-1193998-SI.pdf]

microorganisms

Table S1. List of primers used in this study.

Primer	Primer sequence 5′ → 3′	Target genes	product size	Annealing T	Ref
16S F	AAATTGAAGAGTTTGATCATGG	16Sr DNA	1554pb	55°c	[1]
16S R	GCTTCTTTAAGGTAAGGAGGT				
Carbapenem resistant genes Conventional PCR					
NDM F	GGTTTGGCGATCTGGTTTTC	NDM variants	621pb	60°c	[2]
NDM R	CGGAATGGCTCATCACGATC				
GES F	AGTCGGCTAGACCGGAAAG	GES variants	399pb	58°c	[3]
GES R	TTGTCCGTGCTCAGGAT				
OXA4F	GCTTGATCGCCCTCGATT	OXA-48	281pb	60°c	[3]
OXA4R	GATTTGCTCCGTGGCCGAAA				
IMP F	TTGACACTCCATTTACAG	IMP Variants	139pb	56°c	[3]
IMP R	GATCGAGAATTAAGCCACCC				
VIM F	GATGGTGTTTGGTCGCATA	VIM variants	390pb	60°c	[3]
VIM R	CGAATGCGCAGCACCAG				
KPC F	CATTCAAGGGCTTTCTTGCTGC	KPC variants	538	60°c	[3]
KPC R	ACGACGGCATAGTCATTTGC				
ESBL genes multiplex PCR					
TEM F	CATTTCCGTGTCGCCCTTATTC	TEM-1	800bp	60°c	[3]
TEM F	CGTTCATCCATAGTTGCCTGAC	TEM-2			
SHV F	AGCCGCTTGAGCAAATTAAAC	SHV variants	713bp	60°c	[3]

SHV R	ATCCCGCAGATAAATCACCAC				
CTX F	CGC TTT GCG ATG TGC AG	CTX-M types	550	55°c	[4]
CTX R	ACC GCG ATA TCG TTG GT				
OXA F	GGCACCAGATTCAACTTTCAAG	OXA-1/4/30	564pb	60°c	[3]
OXA R	GACCCCAAGTTTCCTGTAAGTG				

References

1. Ye, H.; Li, Y.; Li, Z.; Gao, R.; Zhang, H.; Wen, R.; Gao, G.F.; Hu, Q.; Feng, Y. Diversified mcr-1-Harbouring Plasmid Reservoirs Confer Resistance to Colistin in Human Gut Microbiota. *mBio* **2016**, *7*, e00177, doi:10.1128/mBio.00177-16.
2. Hatrongjit, R.; Kerdsin, A.; Akeda, Y.; Hamada, S. Detection of plasmid-mediated colistin-resistant and carbapenem-resistant genes by multiplex PCR. *MethodsX* **2018**, *5*, 532-536, doi:10.1016/j.mex.2018.05.016.
3. Dallenne, C.; Da Costa, A.; Decré, D.; Favier, C.; Arlet, G. Development of a set of multiplex PCR assays for the detection of genes encoding important beta-lactamases in Enterobacteriaceae. *The Journal of antimicrobial chemotherapy* **2010**, *65*, 490-495, doi:10.1093/jac/dkp498.
4. Ali, T.; Ur Rahman, S.; Zhang, L.; Shahid, M.; Zhang, S.; Liu, G.; Gao, J.; Han, B. ESBL-Producing Escherichia coli from Cows Suffering Mastitis in China Contain Clinical Class 1 Integrons with CTX-M Linked to ISCR1. *Frontiers in microbiology* **2016**, *7*, 1931, doi:10.3389/fmicb.2016.01931.
